# Auto-Brewery Syndrome After COVID-19 Infection

**DOI:** 10.14309/crj.0000000000001248

**Published:** 2024-02-08

**Authors:** Sarah R. Yates, Akira Saito

**Affiliations:** 1 Department of Internal Medicine, Indiana School of Medicine, Indianapolis, IN; 2 Division of Gastroenterology and Hepatology, Indiana School of Medicine, Indianapolis, IN

**Keywords:** COVID-19, auto-brewery syndrome, gut fermentation syndrome, small intestines, dysbiosis

## Abstract

Auto-brewery syndrome (ABS) is a rare medical condition, wherein gut microbiota ferment carbohydrates to alcohol. Risk factors for ABS include diets high in carbohydrates and sugars, diabetes mellitus, prior gastrointestinal surgery, nonalcoholic fatty liver disease, and certain genetic mutations, among others. We provide a case of ABS that developed after known COVID-19 infection, which may be one of the contributing factors to its development.

## INTRODUCTION

Auto-brewery syndrome (ABS), also known as gut fermentation syndrome and endogenous alcohol fermentation syndrome, is a rare, underdiagnosed, and understudied condition.^1^ It is believed that fungal and/or bacterial dysbiosis in the oral cavity, gastrointestinal, or urinary system results in fermentation of ingested carbohydrates into ethanol, which is subsequently absorbed into the bloodstream.^1–3^ Diagnosis is confirmed with blood alcohol levels after ingestion of a glucose load.^4^ ABS has a significant impact on patient quality of life and negative social and legal consequences. Patients present with a variety of symptoms consistent with intoxication, including but not limited to slurred speech, walking difficulty, disorientation, mood changes, and seizure.^5^ Up to September 2020, only 20 cases of confirmed ABS had been reported in the literature.^5^ In this report, we discuss new-onset symptoms and diagnosis of ABS soon after COVID-19 infection and recovery, which has not been previously reported.

## CASE REPORT

The patient is a 52-year-old man with a medical history of depression who was diagnosed with COVID-19 confirmed through antigen testing. His symptoms were mild upper respiratory symptoms, and he seemingly recovered quickly after diagnosis. However, 1 month later, he began to intermittently experience symptoms of episodic dizziness, slurring of speech, behavioral changes such as getting highly emotional or aggressive, and foggy memory. He presented to the emergency department after a particularly notable episode where he abruptly began shouting and became aggressive during a family dinner. In the emergency department, his blood ethanol level was found to be 212 mg/dL. All other laboratory tests, urine drug screen, and head computed tomography were negative. Although the patient does occasionally drink alcohol, approximately 1–2 times per month in social settings only, he vehemently denied any recent alcohol use before presentation. He has no previous alcohol withdrawal or psychosis and does not use any other illicit substances. Of note, his chronic medication list includes a multivitamin, vitamin D, vitamin C, zinc, an over-the-counter probiotic, and sertraline.

After this event, the patient purchased a breathalyzer and noticed elevated blood alcohol content (BAC) after meals, especially those high in carbohydrates. ABS was suspected, and he was referred for further evaluation. He underwent carbohydrate challenge testing at a motility laboratory. A baseline BAC of 0.0% was obtained through a breathalyzer. The patient then ingested 200 g of glucose in 500 mL of water, after which BAC was measured every 15 minutes for the first hour and then every 30 minutes thereafter. At 83 minutes, the patient became emotional, began to cry, and experienced frequent eye blinking. At this time, his BAC was found to be 0.149% (149 mg/dL). His symptoms continued, and 15 minutes later, his BAC was measured again and found to be 0.046% (46 g/dL). The patient was supervised during the entire examination in our motility laboratory. This confirmed the diagnosis of ABS. He also underwent small bowel enteroscopy and colonoscopy to obtain gastric, small bowel, and colonic fluid aspirate. Endoscopic findings were unremarkable, and fungal cultures were negative. Bacterial cultures were not obtained.

The patient was placed on a strict low carbohydrate diet, started on a daily probiotic, and was treated with daily fluconazole. This reduced the frequency and severity of his episodes. On tapering his fluconazole dosing, his symptoms became more frequent, occurring once or twice per week. He was then started back on daily fluconazole 200 mg with plans to treat for at least 3 months. He was referred to a dietitian for further counselling on strict very low/no carbohydrate diet. He has subsequently been referred to Infectious Disease for further antimicrobial recommendations. In addition, he was counseled on driving restrictions, including checking his BAC with a breathalyzer each time before he operates a vehicle and avoiding ingestion of carbohydrates for at least 2 hours before operating a vehicle.

## DISCUSSION

Endogenous formation of alcohol in the gastrointestinal tract, now known most commonly as ABS, was first presumptively diagnosed in an African child in 1948.^6^ Since that time, several cases of the endogenous ethanol formation have been reported across the world. In addition to endogenous fermentation, genetic mutations in the components of alcohol metabolism may contribute to ABS. Specifically, genetic polymorphisms of alcohol dehydrogenase and aldehyde dehydrogenase 2 may lead to a buildup of ethanol and acetaldehyde.^1^ To our knowledge, this is the first case of ABS diagnosed after COVID-19 infection.

Patients typically present with episodes of alcohol intoxication without alcohol use, but diagnosis of ABS can be challenging because history-taking alone may not differentiate ABS from surreptitious alcohol use. A carbohydrate challenge performed under supervision may reveal a diagnosis of ABS and is therefore recommended in suspected cases. Furthermore, endoscopic aspiration of gastric, small bowel, and colonic secretions has been reported to reveal causative organisms, although this was not the case in our patient. Various yeast species have been implicated in the development of ABS, including those that are fermenting such as *Saccharomyces cerevisiae*, *Saccharomyces boulardii*, and various strains of candida.^5,7–9^ A handful of cases have been linked to bacterial species.^10^

Treatment methods typically include drug therapy, targeted toward the yeast or bacteria that is identified, and dietary modification consisting of high protein and low carbohydrates. The patient's symptoms did improve with fluconazole, and it was recommended to modify his diet. The exact mechanism behind the development of ABS after COVID-19 infection remains unclear. Several studies have been conducted to explore the relationship between gut microbiota and the SARS-CoV-2 virus. Researchers have found that patients with COVID-19 had significant differences in their gut microbiome when compared with those without COVID-19 and that this dysbiosis persisted even after the clearance of the virus.^11^ It is possible that the SARS-CoV-2 virus altered this patient's gut flora enabling an organism capable of endogenous alcohol formation to flourish.

## DISCLOSURES

Author contributions: A. Saito saw the patient in clinic and proposed a case study write up. He additionally assisted in writing and editing the written case report. SR Yates conducted background research as well as wrote and edited the case report. A. Saito is the article guarantor.

Financial disclosure: None to report.

Informed consent was obtained for this case report.
